# First Molecular Evidence of the Presence of Avian Astroviruses in Turkey Flocks of Ecuador Through the Standardization of RT-qPCR Assays Based on SYBR Green

**DOI:** 10.3390/v18030308

**Published:** 2026-03-01

**Authors:** Anthony Loor-Giler, Camila Sanchez-Castro, Silvana Santander-Parra, David Andrade-Ojeda, Byron Puga-Torres, Renán Mena-Pérez, Martin Campos, Antonio Piantino Ferreira, Sabrina Galdo-Novo, Luis Nuñez

**Affiliations:** 1 Laboratorios de Investigación, Dirección General de Investigación, Universidad de las Américas (UDLA), Antigua Vía a Nayón S/N, Quito EC 170124, Ecuador; a.abel.loor.giler@gmail.com; 2 Facultad de Ciencias Veterinarias, Universidad de Buenos Aires, Av. Chorroarín 280, Buenos Aires 1427, Argentina; sgaldonovo@fvet.uba.ar; 3 Facultad de Ingeniería y Ciencias Aplicadas, Carrera de Ingeniería en Biotecnología, Universidad de Las Américas (UDLA), Antigua Vía a Nayón S/N, Quito EC 170124, Ecuador; camila.sanchez.castro285@hotmail.com; 4 Facultad de Ciencias de la Salud, Carrera de Medicina Veterinaria, Universidad de Las Américas (UDLA), Antigua Vía a Nayon S/N, Quito EC 170124, Ecuador; silvanahsp@yahoo.com (S.S.-P.); david.andrade@udla.edu.ec (D.A.-O.); 5 Facultad de Medicina Veterinaria y Zootecnia, Universidad Central del Ecuador, Jerónimo Leyton s/n Gilberto Gatto Sobral, Quito EC 170521, Ecuador; bpuga@uce.edu.ec (B.P.-T.); rpmena@uce.edu.ec (R.M.-P.); 6 Facultad de Industrias Agropecuarias y Ciencias Ambientales, Carrera Medicina Veterinaria, Universidad Politécnica Estatal del Carchi (UPEC), Antisana S/N y Av. Universitaria, Tulcán EC 040102, Ecuador; rolando.campos@upec.edu.ec; 7 Facultad de Ciencias Veterinarias, Universidad Nacional de Rosario (UNR), Boulevard Ovidio Lagos y Ruta 33 Casilda, Santa Fe 2170, Argentina; 8 Laboratory of Avian Diseases, School of Veterinary Medicine and Animal Science, Department of Pathology, University of São Paulo, São Paulo 05508-270, SP, Brazil; ajpferr@usp.br; 9 One Health Research Group, Facultad de Ciencias de la Salud, Universidad de Las Américas (UDLA), Quito EC 170124, Ecuador

**Keywords:** enteric disease, turkey astroviruses, RT-qPCR assays, SYBR Green

## Abstract

Enteric diseases are a significant challenge for the poultry industry, causing substantial economic losses and affecting productivity. Turkey astrovirus (TAstV) types 1 and 2 and avian nephritis virus (ANV) are recognized as viral pathogens contributing with enteric diseases in turkeys, particularly in young poults. These viruses, part of the *Astroviridae* family, are small, round, non-enveloped, positive-sense RNA viruses with high prevalence in turkey flocks. Despite their importance, they had not been identified in Ecuador until now. This study presents the first detection and molecular characterization of TAstV-1, TAstV-2, and ANV in Ecuadorian turkeys using RT-qPCR assays based on SYBR Green, developed and optimized for high sensitivity and specificity. Two hundred intestinal samples were collected from turkeys with enteric disorders, along with fifty cloacal swabs from apparently healthy turkeys in Pichincha Province. The RT-qPCR assays developed demonstrated a limit of detection of one copy of viral genetic material and high repeatability, with inter and intra-assay coefficients of variation below 1%. Based on these tests, TAstV was detected in 93% of turkey samples with gastroenteritis, and none of the samples of the healthy group tested positive, with ANV being the most prevalent, followed by TAstV-2 and TAstV-1. Phylogenetic analysis of the partial *ORF1b* gene confirmed the genetic relationships between Ecuadorian strains and those from other countries, highlighting possible routes of introduction and evolution of the virus. Co-infections with TAstV-2 and ANV were common, while single infections were predominantly caused by ANV. These findings underscore the critical need for surveillance and biosecurity measures to control the spread of these viruses within Ecuador’s poultry industry. This study provides valuable insights on astrovirus presence in Ecuadorian turkey flocks and establishes robust diagnostic tools for monitoring and managing turkey astrovirus infections.

## 1. Introduction

The poultry industry is one of the most important sectors in Ecuador’s agricultural economy, contributing 4% of the country’s total gross domestic product (GDP) and 24% of the specific GDP for the agricultural sector. It generates more than 300,000 job opportunities in the production chain and produces 4 million United States (U.S.) dollars per year. In 2022, 22.753 tons of turkey meat were produced in the country [1]. Thus, turkey breeding plays a very important role in poultry farming due to the generation of high-quality protein, which supplies the national and international market. However, the poultry industry has many challenges, one of which is enteric diseases that represent a significant problem for animal health [2]. Enteric diseases in turkeys have been linked to viral pathogens that are associated with the genesis of these diseases [3]. Among the most prevalent turkey enteric viruses are astrovirus, parvovirus, rotavirus, and coronavirus. Astrovirus is particularly significant in this industry due to its high incidence in turkey flocks and the substantial economic losses associated with its symptoms [4,5].

Astroviruses are small, non-envelope positive-sense RNA viruses; their genome length varies between 6.5 and 7.7 kb [6]. They have a star-like morphology with 5–6 projections present on the surface of the viral capsid [7]. There are three open reading frames (ORFs): ORF1a encoding the non-structural protein, ORF1b encoding RNA-dependent RNA-polymerase, and ORF2 encoding the viral capsid structural protein; these ORFs are flanked by untranslated regions (UTRs), one at the 5′ end and one at the 3′ end, followed by a poly-A tail [8,9]. They are part of the *Astroviridae* family, consisting of the genera *Mamastrovirus* and *Avastrovirus*, which infect mammals and avian species, respectively. The genus Avastrovirus now has seven officially identified species that infect various bird hosts [10,11]. These viruses cause enteric diseases since they are distributed mostly in the intestine, causing poor digestion, hepatitis, nephritis, and deficiency in nutrient absorption [12].

Currently, three types of astroviruses have been identified that infect turkeys: turkey astrovirus 1 (TAstV-1), turkey astrovirus 2 (TAstV-2), and avian nephritis virus (ANV) [13]. TAstV-1 was first detected in the United Kingdom in 1980 in turkeys showing diarrhea and high mortality [14]. Later, in 2000, TAstV-2 was identified and is associated with poult enteritis complex (PEC) in the U.S. [15]. Additionally, ANV has been reported in turkeys with enteritis, a virus mainly associated with chickens, but it can also infect other avian species, such as turkeys, ducks, geese, and pigeons, among others, where it is mainly associated with tubulonephrosis, visceral gout, diarrhea, and growth retardation [16].

The poult enteritis complex (PEC) is a pathological condition present in young turkeys characterized by diarrhea, immunosuppression, depression, enteritis, weight loss, feed conversion deficiency, and mortality, whereas when astroviruses infect turkeys, not all symptoms may be present simultaneously [17]; this is attributed to different etiological agents, including a range of viruses such as TAstV, and certain bacterial pathogens [18]. Several syndromes have been reported as part of this complex: poult enteritis and mortality syndrome (PEMS), characterized by diarrhea, lack of growth, and high mortality in young turkeys; runting–stunting syndrome (RSS), which results in growth and development below what is expected for their age; light turkey syndrome (LTS), characterized by reduced body weight; and finally poult enteritis syndrome (PES), which involves inflammation and irritation of the intestine in poults, resulting in diarrhea and weight loss [19].

The application of molecular techniques for the detection of infectious agents has proven to be highly effective in terms of speed, sensibility, and specificity, surpassing traditional methods such as electron microscopy, cell culture, and immunoassays [20]. These approaches allow for the identification of viral presence even in samples with low viral loads. RT-PCR assays demonstrate a high degree of sensitivity and specificity for detecting viral RNA, establishing them as invaluable tools for the diagnosis and surveillance of viral infections [21,22].

Turkey astroviruses (TAstV-1, TAstV-2, and ANV) have been well-documented in avian populations in several countries [14,17,18,23,24,25]. However, no evidence of these viral agents has been reported in Ecuador, leaving a significant gap in our understanding of their epidemiological status within the country, although reports of turkeys affected with enteric disease are reported by poultry veterinarians, where bacteria and parasites have not been diagnosed (not published data). This lack of information has a detrimental effect on the insights available on the prevalence, geographic distribution, and burden of disease in the national poultry sector. The substantial economic impact of astroviruses in turkeys, due to high morbidity, clinical severity, and production losses, renders the establishment of baseline data on the presence of these pathogens in Ecuador imperative.

The aim of this study is to address this gap by providing the first molecular report of TAstV-1, TAstV-2, and ANV in turkeys from Ecuador. This study contributes to a broader understanding of the presence of these viruses in this territory as well as their potential impact on poultry health and productivity. In addition, it contributes a diagnostic tool that could be used in the diagnosis of astrovirus in turkeys.

## 2. Materials and Methods

### 2.1. Sampling

In this study, a total of 250 samples were collected, including 200 turkey (1–110 days old) intestinal tracts from dead turkeys with enteric diseases and cloacal swabs from 50 young turkeys (7 days old) without signs of intestinal disease as a control group. Cloacal swabs were selected for clinically healthy birds because they allow non-invasive sampling under routine farm conditions, whereas intestinal tissues were obtained from deceased turkeys with enteric disease since intestinal content generally provides higher viral loads and improves diagnostic sensitivity for enteric viruses such as astrovirus. Sampling intestinal tissue from live birds was not feasible due to animal welfare and farm biosecurity constraints. All samples were obtained from 10 turkey farms in the province of Pichincha in Ecuador. The poultry farmers reported that samples from dead animals were maintained at approximately 4 °C immediately after collection; this refrigeration was intended only for temporary preservation during on-farm handling and transport to limit nucleic acid degradation. Upon arrival at the research laboratory, samples were stored at −20 °C for short-term preservation of viral genetic material until molecular analysis. Clinical records indicated that the animals died as a consequence of previously observed enteric disease on the farm (Supplementary Material, Table S1). For this study, deceased turkeys of 1 to 110 days of age with clinical records indicative of enteric disease, such as apathy, ruffled feathers, diarrhea, and cloacal pasting, were sent to the research laboratories of the Universidad de Las Americas to detect the presence of enteric viruses. The intestines (jejunum) were used to determine the presence or absence of turkey astrovirus (TAstV-1, TAstV-2, and ANV) as well as for the standardization and validation of the RT-qPCR assay based on SYBR Green chemistry. All procedures conducted in the present investigation were in accordance with the guidelines and the approval of the Committee on the Care and Use of Laboratory and Domestic Animal resources of the Agency of Regulation and Control of Phytosanitary and Animal Health of Ecuador (AGROCALIDAD), under number #INT/DA/019 (31 January 2018).

### 2.2. Nucleic Acid Extraction

Intestinal samples were dissected to obtain a 0.5 × 0.5 cm^2^ portion of jejunal tissue of approximately 100 mg and suspended in 250 µL of phosphate buffer saline (PBS) to maintain the integrity of the biological material. Cell lysis was performed using stainless steel beads in TissueLyser (QIAGEN, Hilden, Germany); the macerated tissue was used for viral RNA extraction using TRIzol Reagent (Invitrogen by Life Technologies, Carlsbad, CA, USA) according to the manufacturer’s instructions for RNA isolation. The cloacal swabs were suspended in 1 mL of PBS and subjected to heat shock following previously described protocols [3]. After centrifugation at 12,000 rcf, 250 μL of the supernatant was collected for viral RNA extraction using TRIzol Reagent (Invitrogen by Life Technologies, Carlsbad, CA, USA). Approximately 1 µg of RNA was converted into cDNA using SuperScript IV Reverse Transcriptase (Invitrogen™ Van Allen Way, Carlsbad, CA, USA) according to manufacturer’s instructions. DNA extraction for positive controls (TuPV and ChPV) used to determine the specificity of the assay was extracted using TRIzol extraction DNA method according to the manufacturer’s instructions.

### 2.3. Hemi-Nested PCR

A pan-astrovirus detection assay, capable of detecting astroviruses across multiple species [26], was applied to the cDNA of 200 turkey jejunum samples to determine the presence of turkey astrovirus genetic material. A fragment of approximately 400 bp of the ORF-1b gene was amplified using the kit Platinum Taq DNA polymerase (Invitrogen by Thermo Fisher Scientific, Carlsbad, CA, USA) for the application of a hemi-nested PCR following the published instructions for an accurate detection of astroviruses [26].

### 2.4. Sequencing and Phylogenetic Analysis

Of the obtained amplicons (40) with a strong intensity of the band obtained from hemi-nested PCR described above were purified with the ExoSap-IT™ Express PCR Product Cleanup System (Applied Biosystems, Santa Clara, CA, USA) according to the manufacturer’s protocol. Subsequently, the purified products were sequenced in both forward and reverse directions using the Big Dye^®^ Terminator v3.1 Cycle Sequencing Kit (Thermo Fischer Scientific), and the sequencing data were generated on the 3500 Series Genetic Analyzer (Applied Biosystems, Foster city, CA, USA). Electropherograms were processed using the Geneious Prime software package, version 10.2.3 (https://www.geneious.com, accessed on 16 January 2026) (Biomatters Ltd., Auckland, New Zealand). The sequences obtained were individually aligned to TAstV-1, TAstV-2, and ANV reference sequences reported in other countries and randomly retrieved from the NCBI GenBank database through Multiple Sequence Alignment using the Clustal Omega 1.2.2 package in the Geneious 10.2.3 software (https://www.geneious.com). Nucleotide similarities were then calculated using the Geneious 10.2.3 software (https://www.geneious.com, accessed on 16 January 2026). Prior to phylogenetic analysis, identical sequences were removed, and turkey coronavirus (TCoV) was used as the outgroup. A phylogenetic tree was inferred with the maximum likelihood statistical method, employing a Tamura–Nei substitution model and bootstrap phylogeny testing with 1000 replicates, as implemented in MEGA 11 software packages [27].

### 2.5. Primers Design for Specific Detection of TAstV-1 and TAstV-2

Specific primers were designed for the detection of TAstV-1 and TAstV-2. Complete genome sequences and *ORF1b* gene sequences of both genotypes of turkey viruses were obtained from the NCBI GenBank database (NC_002470, Y_15936, KU_711025, KT_355319, KC_967118, JX_985653, HQ_317717, KC_967120, JX_083371, JQ_692622, JN_048385, HQ_317733, GQ_301011, FJ_693666, EU_165345, EU_143851, EU_143850, DQ_381388, and NC_005790) and the sequences obtained with the hemi-nested PCR assay carried out in this study. Sequences were aligned with the Geneious Prime^®^ 2022.2.2 software (https://www.geneious.com) in the Clustal Omega package to target amplification of a conserved fragment of the *ORF-1b* gene of each virus. The primers used in this study to amplify a fragment of ANV were originally developed for a hydrolysis probe RT-qPCR assay and were subsequently adapted for use in a two-step RT-qPCR assay in this study with SYBR Green chemistry (Table 1).

### 2.6. Standard Curve Construction

For the standard curve construction, amplification products obtained through hemi-nested PCR and confirmed by sequencing to belong to TAstV-1 and TAstV-2 obtained in Section 2.3 and Section 2.4 were used as positive controls. For ANV, an end-point PCR was applied using the same primers proposed for the RT-qPCR assay. The amplified target fragment for each virus was cloned separately into the TOPO™ TA vector (Thermo Fischer Scientific) and transformed into TOP10 chemically competent *E. coli* cells (Thermo Fischer Scientific). Colony PCR was performed to identify clones containing the vector with the fragment of interest. Plasmid DNA was extracted from the confirmed clones using the PureLink™ Quick Plasmid Miniprep Kit (Invitrogen by Thermo Fisher Scientific, Vilnius, Lithuania) and quantified with a NanoDrop™ 2000 (Thermo Fischer Scientific, Wilmington, DE, USA). The DNA concentration was entered into the DNA Copy Number and Dilution Calculator web tool (Thermo Fischer Scientific) to determine the number of copies of the target sequence. A stock solution containing 10^8^ copies was prepared, followed by ten-fold serial dilutions down to one copy for standard curve construction. A separate standard curve was generated for each virus to ensure accurate quantification in the RT-qPCR assays.

### 2.7. RT-qPCR Assay

For each virus, a separate two-step RT-qPCR, singleplex, was performed using PowerUp SYBR Green Master Mix (Applied Biosystems by Thermo Fisher Scientific, Vilnius, Lithuania) and specific primers for each virus (Table 1). The reaction was prepared with 1 µL of UltraPure™ DNase/RNase-Free Distilled Water (Invitrogen by Thermo Fisher Scientific, Carlsbad, CA, USA), 5 µL of PowerUp SYBR Green Master Mix 1X, 0.75 µM of each primer (2 per virus) for TAstV-1 and TAstV-2, 0.60 µM of each primer for ANV, and 2.5 µL of cDNA. Primer concentrations were empirically optimized (0.60–0.75 μM) to achieve maximal analytical sensitivity while avoiding primer–dimer formation and non-specific amplification, as assessed by melt curve analysis. For each virus, the standard cycling mode for PowerUp SYBR Green was applied, which included an initial UDG inactivation step at 50 °C for 2 min, denaturation at 95 °C for 2 min, followed by 45 cycles of denaturation at 95 °C for 10 s, an annealing step at 56 °C for 30 s, and an extension step at 72 °C for 30 s. As positive controls, a point from the standard curve was included for each virus, with ddH_2_O used as negative control and a non-template control (NTC). A melting curve analysis was performed for every run, and all samples were analyzed in duplicate to ensure consistent results.

### 2.8. Analytical Sensitivity of the RT-qPCR Assay

The limit of detection (LoD) and limit of quantification (LoQ) were defined as the lowest concentration at which consistent amplification was achieved in the ten serial dilutions of the standard curve, thus establishing the minimum threshold of the method for reliable detection and quantification of the target sequence. Analytical sensitivity was initially evaluated using a synthetic construct, determining a detection threshold as low as 1 copy/μL. To evaluate the performance of the assay in a biological context, the constructed curve was also tested in RNA samples extracted (and subsequently cDNA) from jejunal tissue, and detection remained at the same limit, indicating its robustness even in the presence of host-derived RNA.

### 2.9. Analytical Specificity of the RT-qPCR Assays

The specificity of the RT-qPCR assay was determined using positive controls for TuPV, ChPV, and CAstV; positives controls correspond to the isolated viral agents previously described [29,30,31]. The positive controls (TuPV, ChPV, and CAstV) underwent the RT-qPCR protocols devised in this study, following the previously established conditions.

### 2.10. Analytical Repeatability of the RT-qPCR Assays

To ensure the repeatability of the assay, both inter-assay and intra-assay were evaluated using tenfold serial dilutions ranging from 10^7^ to 10^3^ copies of DNA previously prepared for the standard curve; each of these dilutions were aliquoted and stored at −20 °C until use. This process was assessed by calculating the average cycle. For the inter-assay evaluation, aliquots of each dilution were amplified under identical conditions across five independent runs conducted in different days. Intra-assay repeatability was analyzed with five replicates of each dilution, with the RT-qPCR assay performed in a single run. The repeatability was quantified by calculating the average Cq values and the coefficient of variation (CV) for both inter-assay and intra-assay measurements. Stability of the RT-qPCR assay was further assessed through CV values, with lower CV percentages indicating higher assay reliability. All procedures followed established guidelines [32].

### 2.11. Validation Metrics

The diagnostic method proposed in this study was validated following the recommendations outlined in the MIQE guidelines and the WOAH Terrestrial Manual (Chapter 1.01.06), providing the required data for Stage 1 validation [32,33]. To assess the reliability of the test, diagnostic performance indicators were calculated using the following equations:

Diagnostic performance metrics were estimated using standard epidemiological formulas. Sensitivity was calculated as the proportion of confirmed positives correctly identified by the assay and expressed as TP divided by the sum of TP and FN, whereas specificity corresponded to TN divided by TN plus FP, representing the ability to correctly classify negative samples.

The positive predictive value (PPV) was derived from the relationship between test sensitivity and the expected prevalence of infection in the evaluated population, following the expression [sensitivity × prevalence]/{[sensitivity × prevalence] + [(1 − specificity) × (1 − prevalence)]}. In parallel, the negative predictive value (NPV) incorporated test specificity and the probability of true negatives in the population using the formula [specificity × (1 − prevalence)]/{[specificity × (1 − prevalence)] + [(1 − sensitivity) × prevalence]}.

These formulas were applied according to Chapter 6 of Clinical Epidemiology by Fletcher [34]. In this context, TP refers to true positives, FN to false negatives, TN to true negatives, and FP to false positives.

### 2.12. Statistical Analysis

Descriptive statistics were conducted on the analyzed samples using a dataset that included age (days), viral copies, and RT-qPCR results for each virus (TAstV-1, TAstV-2, and ANV). The distribution of the samples was assessed using the Shapiro–Wilk test. Cohen’s Kappa test was performed to obtain the kappa coefficient and analyze the agreement between the two molecular methods used in this study. A Kruskal–Wallis test was then applied to determine whether significant differences existed among the three viruses regarding the distribution of viral loads detected by RT-qPCR. All tests were conducted with a 95% confidence interval in RStudio 2025.09.2.

### 2.13. Genbank Accesion Numbers

The partial *ORF1b* gene sequences obtained in this study have been submitted to GenBank and are available under the following accession numbers: ANV: PV568354 (26T), PV568355 (39T), PV568356 (200T), PV568357 (196T), PV568358 (191T), PV568359 (113T), PV568360 (101T), PV568361 (133T), PV568362 (74T), PV568363 (23T), PV568364 (31T), PV568365 (166T), PV568366 (25T), PV568367 (28T), and PV568368 (21T); TAstV-1: PQ998992 (33T), PQ998993 (2T), and PQ998994 (3D ECU); TAstV-2: PV568350 (78T), PV568351 (16T), PV568352 (72T), and PV568353 (52D ECU).

## 3. Results

### 3.1. Standard Curves

**Figure 1:**
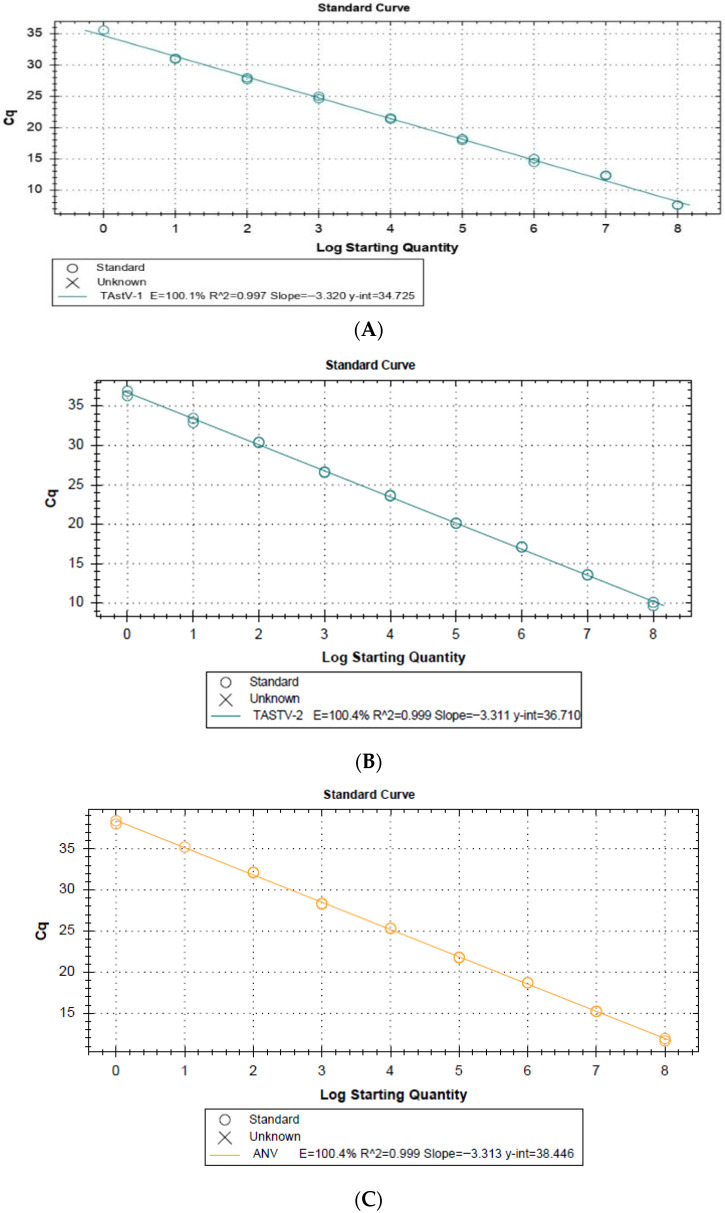

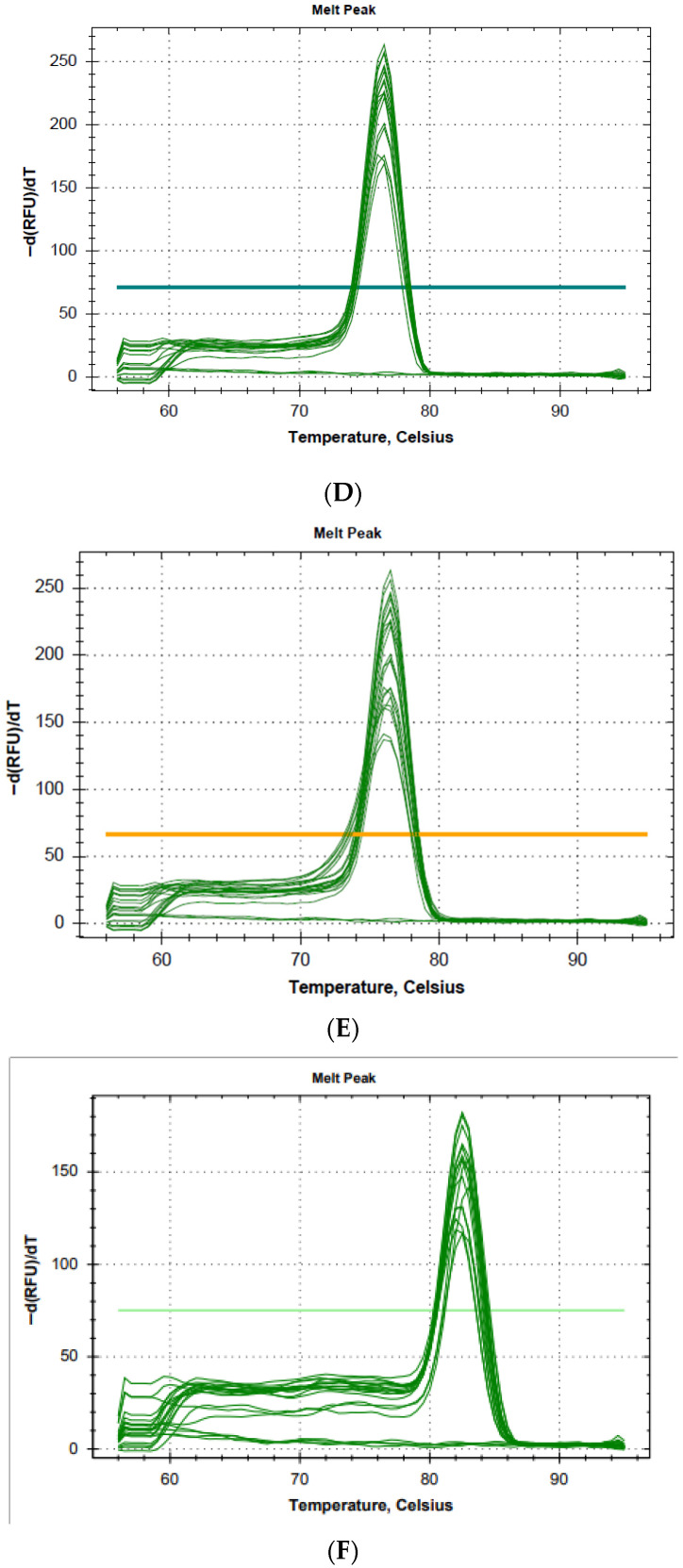
Standard curves generated for the detection of turkey astroviruses, (**A**) TAstV-1, (**B**) TAstV-2, and (**C**) ANV. Melt curves (peaks) for qPCR assays, (**D**) TAstV-1, (**E**) TAstV-2, and (**F**) ANV.

Standard curves generated from ten-fold serial dilutions for the detection assays of TAstV-1, TAstV-2, and ANV showed an efficiency of 100.1% (Figure 1A), 100.4% (Figure 1B), and 100.4% (Figure 1C), respectively. All amplified points showed a melting curve corresponding to the fragment of interest (ANV (82.5 °C), TAstV-1 (76.5 °C), and TAstV-2 (76 °C)). The assay demonstrated high specificity where no negative controls, dimers, or non-specific products were amplified in any run. The limit of detection (LoD) and limit of quantification (LoQ) for each assay were determined, demonstrating that both were up to 10^0^ copies of genetic material in each assay. The maximum Cq to consider a sample positive was 36, 37, and 39 for TAstV-1, TAstV-2, and ANV, respectively.

### 3.2. Specificity Analysis of the RT-qPCR Assays

The specificity assessment of the RT-qPCR assay for TAstV-1, TAstV-2, and ANV detection exclusively demonstrated amplification curves for samples positives for the viruses mentioned above. No amplification was detected in any of the other viruses: TuPV, ChPV, or CAstV.

### 3.3. Diagnostic Test Performance Metrics

Based on the evaluation of the proposed diagnostic assay using established performance metrics, the method demonstrated sensitivity and specificity levels of 100% within the analyzed sample set. Furthermore, both the negative predictive value (NPV) and positive predictive value (PPV) were calculated at 100%. While these findings indicate excellent diagnostic agreement under the conditions of this study, such estimates should be interpreted cautiously pending validation in larger and more diverse cohorts.

### 3.4. Repeatability Analysis

The repeatability assay, performed using ten-fold standard serial dilution from 10^7^ to 10^3^ copies, showed the following inter-assay and intra-assay coefficients of variation for each virus: for TAstV-1, the inter-assay coefficient of variation ranged from 0.09% to 0.25%, while the intra-assay coefficient of variation ranged from 0.17% to 0.39%. TAstV-2 exhibited inter-assay coefficient of variation values between 0.20% and 0.99%, with an intra-assay coefficient of variation ranging from 0.17% to 0.96%. For ANV, the inter-assay coefficient of variation ranged from 0.30% to 0.65%, and the intra-assay coefficient of variation ranged from 0.40% to 0.90% (Table 2).

### 3.5. Detection of Turkey Astroviruses

In intestinal samples from turkeys with enteric disease, the pan-astrovirus end-point hemi-nested PCR assay detected the presence of astrovirus in 109 samples (54.5%), while the RT-qPCR assay developed in this study identified the presence of any astrovirus that infects turkeys in 186/200 samples, corresponding to 93% of the total analyzed samples. The presence of astrovirus was not identified in the cloacal swabs from the control group (apparently healthy turkeys) using any method. Cohen’s Kappa coefficient was κ = 0.078, which indicates only a slight agreement between the two analyzed methods. This low concordance reflects that the RT-qPCR assay detected a substantially higher number of positive samples compared to the end-point PCR method. In addition, by RT-qPCR assay, specific astroviruses were detected: 9 samples (4.5%) were identified as TAstV-1, 75 samples (37.5%) were identified as TAstV-2, and 178 samples (89%) were identified as ANV. The results of the Shapiro–Wilk test indicated that the dataset did not follow a normal distribution (*p* < 0.05). According to the Kruskal–Wallis test, no statistically significant differences were observed in the distribution of positive results among the three viruses analyzed (*p* > 0.05), suggesting a similar occurrence of TAstV-1, TAstV-2, and ANV in the sampled animals.

#### 3.5.1. Distribution of Astroviruses by Age in Turkeys with Enteric Disease

The detection of TAstV-1, TAstV-2, and ANV varies across different age groups. Among poults aged ≤10 days, a low presence of astroviruses was observed, with only one sample testing positive for TAstV-1 and TAstV-2 and six samples positives for ANV, indicating that ANV was the predominant virus in this age group. In the age group between >10 to ≤90 days, a substantial increase in viral detection was noticed, with 4 positive samples for TAstV-1, while TAstV-2 was detected in 59 samples and ANV in 119, highlighting that ANV was again the most frequently detected virus. Finally, for turkeys aged >90 days, the presence of TAstV-1 remained low (4 positive samples), while the detection rates for TAstV-2 and ANV were 15 and 53 positive samples, respectively (Table 3). ANV showed statistically significant differences; however, given the disparity in samples between groups, the analysis may have been biased.

#### 3.5.2. Quantification of Viral Agents in Turkeys with Enteric Disease

The RT-qPCR assay was capable to quantify viral loads in each turkey astrovirus (TAstV-1, TAstV-2, and ANV)-positive samples in this study. Among age groups, the highest average viral load was exhibited in turkeys aged >90 days, with TAstV-2 and ANV displaying particularly elevated levels, while the lowest viral loads were found in turkeys aged ≤10 days. However, for TAstV-1, the highest average of GCs/mg (mg of tissue) was found in individuals aged ≤10 days, and the lowest viral loads were from turkeys of >90 days of age (Table 4). No significant differences were found.

#### 3.5.3. Co-Infection Analysis in Turkeys with Enteric Disease

Of the 186 positive samples, co-infections patterns were identified as follows: no samples tested positive for the co-infection of TAstV-1 and TAstV-2 (without ANV). Five samples (2.7%) were positive for the co-infection of TAstV-1 and ANV, while sixty-three samples (33.9%) were positive for the co-infection of TAstV-2 and ANV. Additionally, four samples (2.1%) showed co-infection with all three turkey astroviruses (TAstV-1, TAstV-2, and ANV). For single infections, ANV presented the highest number of positives with 106 samples (53%), while 8 samples (4%) were only infected with TAstV2, and no samples were positive just for TAstV-1 (Table 5). Single infection by ANV showed significant differences.

### 3.6. Phylogenetic Analysis

**Figure 2:**
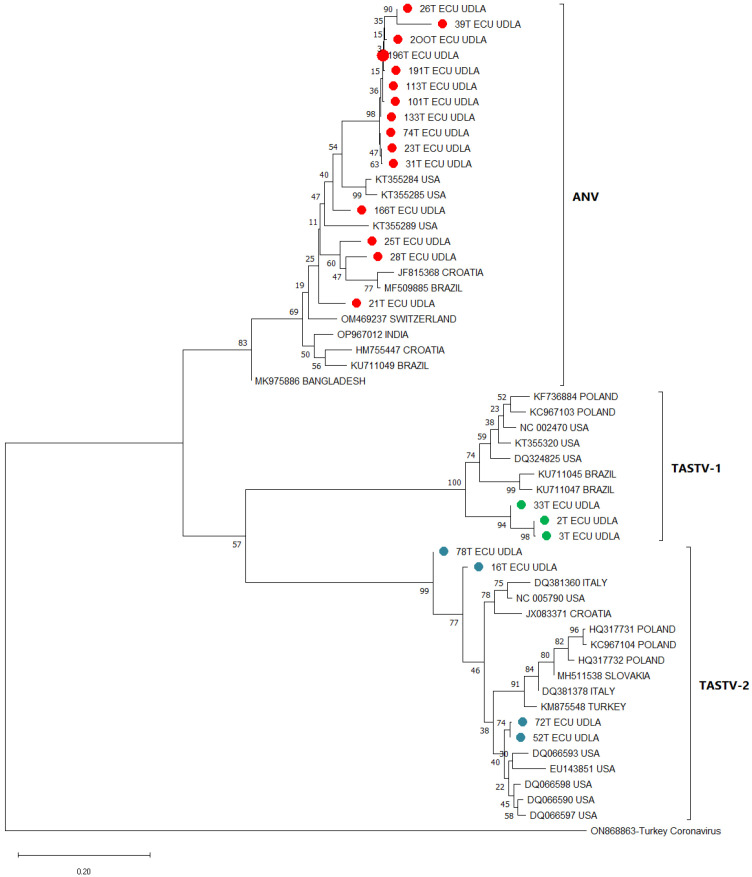
Phylogenetic analysis of TAstV isolates based on partial *ORF1* gene fragment sequences generated in the present study, together with reference sequences retrieved from GenBank and organized by their country of origin. Multiple sequence alignment was performed using the Clustal Omega package implemented in Geneious Prime v2.0.10 (https://www.geneious.com). The phylogenetic tree was inferred with the MEGA 11 software package, applying the maximum likelihood method under the Tamura–Nei substitution model. Bootstrap support values (1000 replicates) are displayed along the branches. The scale bar indicates the number of substitutions per nucleotide site. Sequences obtained in this work are highlighted with circles, ANV sequences are depicted in red, TAstV-1 sequences in green, and TAstV-2 sequences in blue.

The assembled phylogenetic tree revealed different clades grouping the sequences obtained in this study for TAstV-1, TAstV-2, and ANV, corresponding to the species *Avastrovirus 1*, *Avastrovirus 3*, and *Avastrovirus 2*, respectively, along with sequences retrieved randomly from the NCBI GenBank database (Figure 2). Three main clades were formed, corresponding to TAstV-1, TAstV-2, and ANV, each supported by a bootstrap value of 100%, 99%, and 83%, respectively, indicating that each clade corresponded to a specific type of astrovirus. The ANV sequences obtained in this study were distributed across several branches, with some clustering closer with sequences from United States (166T), and others grouped closely with sequences from Croatia and Brazil (28T and 25T). Additionally, the ANV sequence 21T clustered closely with a sequence from Switzerland. The TAstV-1 sequences from this study all grouped together within a single clade, suggesting high genetic similarity among these sequences; this clade had a close phylogenetic relationship with TAstV-1 sequences from Brazil. The four sequences of TAstV-2 obtained in this study were divided into two groups. Three of the sequences clustered together in a single clade, while the remaining sequence (78T) formed a separate branch.

The nucleotide matrix (Table 6) showed high NT similarity between the Ecuadorian TAstV-1, TAstV-2, and ANV sequences and other sequences retrieved from the NCBI. TAstV-1 sequences obtained in this study showed a 100% NT similarity between them and showed high similarity with sequences from other countries, with an NT similarity ranging from 86.8 to 87.3% with sequences from Brazil and an NT similarity of 84.2% with a sequence from Poland. Ecuadorian TAstV-2 sequences revealed an NT similarity of 98.6–100% between them with sequences reported in the NCBI, and we also found an NT similarity of 86.5–93.4% with sequences from USA, an NT similarity ranging from 87.5% to 87.8% with Croatia sequences, an NT similarity of 86.5–87.8.% with sequences from Italy, an NT similarity of 88.6% with Turkey sequences, an NT similarity of 88.2–88.9% with Poland sequences, and an NT similarity ranging from 88% to 89.3% with Slovakia sequences. Ecuadorian ANV sequences demonstrated an NT similarity ranging from 80.9% to 92.8% between them, and they also showed high NT similarity with sequences from Croatia, Bangladesh, Switzerland, India, Brazil, and the United States, showing ranges of NT similarity of 77.2–89.4%, 80.3–89.4%, 81.3–90.5%, 80.4–91.3%, 79.1–89.5%, 77.2–91%, and 82.2–90.5%, respectively.

## 4. Discussion

As previously mentioned, astroviruses are known as one of the causative agents of enteritis and increased mortality in avian species such as turkeys, chickens, and guinea fowl [24]. In agreement with earlier reports, in turkey flocks, these viruses produce the poult enteritis complex (PEC), which includes syndromes like PEMS, PES, LTS, and RSS [35], leading to an economic impact on poultry industries [36]. In this study, astrovirus detection was assessed using two molecular approaches: a previously described hemi-nested PCR and the novel RT-qPCR assays developed here. The RT-qPCR assays demonstrated superior performance not only by detecting a larger proportion of positive samples but also by differentiating among TAstV-1, TAstV-2, and ANV, whereas the hemi-nested PCR lacked specificity and amplified diverse astrovirus types indiscriminately. This discrepancy is consistent with the low Cohen’s Kappa coefficient (0.078), which indicates poor agreement between the methods and highlights the enhanced diagnostic capacity of RT-qPCR. Furthermore, the assays demonstrated high analytical sensitivity, being capable of detecting as little as a single copy of viral genetic material (Figure 1), and high specificity with no amplification of other enteric viruses, which is further supported by the negative results obtained in the 50 cloacal swabs from apparently healthy turkeys included as a control group; this was repeatable, as inter- and intra-assays indicate values lower than 10% (Table 2). Compared to previously reported studies, the present assays surpassed others [37,38,39] in sensitivity. For instance, the multiplex RT-qPCR released in 2005 [37] demonstrated an LoD of 100–600 copies for TAstV- 2 and TCoV, while this study achieved a lower detection limit capable to identifying as few as one copy of viral genetic material, representing an improvement in sensitivity. Similarly, an end-point multiplex RT-PCR targeting TAstV-1, TAstV-2, ANV, and two other enteric viruses [38] showed limit of detections of 1.33 × 10^4^, 1.98 × 10^5^, and 4.22 × 10^5^, respectively; these values are higher than the ones obtained in this study, underscoring the superior sensitivity of the assays used in this study. Likewise, another RT-qPCR for ANV and CAstV [39] described LoDs of 160 copies yet failed to match the accuracy achieved by this study. A critical advantage of the present assay is its superior detection limits, which enhances the ability to identify infections with low viral loads, such as those during early stages or in latent infections [40]; these characteristics reduce the likelihood of false negatives. These assays target all three types of astroviruses that infect turkeys and do not amplify other enteric viruses, improving diagnostic accuracy. The SYBR Green method used in this study also proved cost-effective compared to hydrolysis probe-based assays [41], making it an accessible option for routine diagnostics.

The present study revealed a high presence of astroviruses, with 93% of the samples of turkeys with enteric disease testing positive for at least one of the target viruses: 4.5% for TAstV-1, 37.5% for TAstV-2, and 89% for ANV. These results differ from the findings of a previous study performed in the United States [38], where 53.12% of samples tested positive for TAstV-1, 71.8% for TAstV-2, and only 21.87% for ANV. The higher presence of ANV in Ecuador compared to the United States may indicate differences in farming practices or biosecurity measures. The practice of chicken farming in Ecuador predates turkey farming, reflecting its earlier establishment and development within the Ecuadorian poultry industry, which implies that as a chicken-origin virus, ANV has been present and circulating within the territory earlier than turkey-origin viruses (TAstV-1 and TAstV-2). Similarly, in 2011, a study performed in Poland [42] reported the presence of turkey astroviruses in analyzed samples: 17.6% for TAstV-1, 58.8% for TAstV-2, and 0% for ANV. The absence of ANV in Poland contrasts with its higher presence in Ecuador, suggesting regional variations in virus circulation, potentially driven by different host population or environmental reservoirs. Consistent with the background outlined earlier, in South America, limited studies have been conducted on turkey astroviruses, as they have only been reported in Brazil [43,44,45,46]. One study reported the presence of TAstV-1 and ANV [44] in 6/60 and 21/60 samples, respectively, while TAstV-2 was not evaluated. Another Brazilian study in 2008 [17] identified both TAstV-1 and TAstV-2 in young turkey flocks with enteric disorders. These findings suggest that turkey astroviruses are established in certain South American regions, but studies for their detection have not been performed yet. The high presence of these viruses in Ecuador emphasizes the need for surveillance protocols to mitigate the spread of this virus in poultry industries. Nevertheless, since sampling targeted clinically affected birds and intestinal tissues, the observed detection frequency should not be interpreted as population-level prevalence. Although statistically significant differences were observed for some parameters, the relatively limited sample size and possible lack of representativeness across groups may affect the robustness of these findings. Consequently, these results should be interpreted cautiously, and confirmation through larger, more diverse cohorts is warranted [34].

Age-related patterns in viral loads were also observed in this study, with young turkeys (1–10 days) exhibiting the highest average of viral loads for TAstV-1; similar characteristics were reported in Poland [42], where younger turkeys were more susceptible to astrovirus infections, potentially due to immunological immaturity [47] and the early contamination related to vertical transmission [48]. Meanwhile, older turkeys showed higher viral loads for TAstV-2 and ANV. Although turkey astrovirus is predominantly associated with young poults, longitudinal studies in commercial flocks have demonstrated continued viral circulation and intermittent detection until slaughter age, suggesting endemic flock persistence rather than age-limited infection [38,49]. These variations could be influenced by flock management practices or exposure to environmental reservoirs [50]. The presence of astroviruses in adult turkeys raises concerns of horizontal transmission, which may occur via direct contact, contaminated surfaces, or shared water sources [51], as well as the potential role of wild birds such as pigeons or insect vectors in introducing the virus to flocks [52]. ANV and TAstV-2 co-infection was the most common finding in this study, with 63 out of 200 samples of affected turkeys testing positive for both viruses simultaneously. A study performed in the United States [53] revealed the most frequent astrovirus detected was TAstV-2, and TAstV-1 and ANV were less detected, but both were always co-infected with TAstV-2, following the same trend as in this study, where the more detected virus (ANV) was co-infected with both TAstV-1 and TAstV-2. These co-infections patterns suggest potential interactions between these viruses, in which infection with one agent may induce local or systemic immunosuppression and thereby enhance susceptibility to secondary viral infections [54,55] The absence of single TAstV-1 infections in this study should be interpreted cautiously. While it may suggest that TAstV-1 frequently occurs in co-infections or may not be the primary etiological agent under the conditions evaluated, this observation cannot exclude its independent pathogenic potential [42,56]. Controlled experimental studies would be required to determine whether TAstV-1 alone can establish infection and induce clinical disease, as has been demonstrated for TAstV-2 and other avian astroviruses in experimental settings [57,58].

The phylogenetic analysis executed with a fragment of the *ORF1b* gene revealed the presence of TAstV-1, TAstV-2, and ANV in Ecuadorian turkey flocks. As commonly described in astrovirus molecular studies, this marker is widely used for initial molecular typing because it is conserved and allows discrimination at the species or genogroup level, although it provides limited resolution for strain-level epidemiology [38,56]. The ANV sequences were distributed across several branches, with some clustering closely to sequences from the United States, while others grouped with sequences from Croatia and Brazil, indicating a diverse presence in different regions. The TAstV-1 sequences obtained in this study all clustered together within a single clade, showing high genetic similarity, and they were closely related with sequences from Brazil. Previous studies have indicated that TAstV-1 typically exhibits lower overall genetic diversity at the global scale, often forming compact and well-supported clades, whereas TAstV-2 displays greater heterogeneity across continents [23,42]. However, the clearly defined clades observed in this study for both TAstV-1 and TAstV-2 are consistent with localized circulation patterns, where regional lineages can appear highly structured when sampling is restricted to a single production system or geographically close flocks. This situation has also been described in other country-level studies of turkey astroviruses [24,59]. The TAstV-2 sequences were divided into two groups, with three clustering together in a single clade and one close to sequences from the USA. This pattern aligns with earlier reports describing TAstV-2 as a genetically more variable species, often forming multiple subclusters across continents [55,60]. This highlights the need for further research analysis with the variable region of the astrovirus genome (ORF2), which contains the hypervariable region used to define genotypes, antigenic variants, and lineage-specific markers, to understand the circulating strains present in Ecuador and their interactions with other viruses [56,61]. Full-genome sequencing approaches are also recommended to detect recombination events, which are well-documented in avian astroviruses and contribute to their evolutionary dynamics and pathogenic diversity [10,56,62]. Future studies should analyze the complete genome and explore potential recombination events among these viruses, as recombination in the capsid gene has been reported as a key mechanism shaping astrovirus diversity, particularly in TAstV and ANV. Notwithstanding the merits of the present study, it is incumbent upon us to acknowledge its limitations, including the clinically targeted sampling design, the use of different sample matrices for diseased and control birds, the relatively limited cohort size, and the restricted cross-reactivity panel, all of which should be considered when interpreting prevalence estimates and assay performance.

## 5. Conclusions

This study demonstrates for the first time the presence of turkey astroviruses (TAstV-1, TAstV-2, and ANV) in Ecuadorian turkey flocks, with ANV being the most frequently detected virus. The findings reveal age-related differences in viral loads and a high occurrence of co-infections, particularly between ANV and TAstV-2, suggesting potential interactions that may influence disease dynamics. The RT-qPCR assays developed in this study showed superior sensitivity, specificity, and diagnostic accuracy compared to hemi-nested PCR and previously reported molecular methods, making them a reliable tool for astrovirus detection. These results underscore the need for continuous surveillance and further research on the role of these viruses in poultry health and production.

## Figures and Tables

**Table 1:** Primers used in this study.

Primers	Target	Assay	Sequence (5’-3′)	Length	Reference
TAstV1-F-DP	ORF1b	RT-qPCR	AGAACACAAYAGGYTACCCAC	60 bp	This study
TAstV1-R-DC			TAACYGCAGCAGTATCATAGGAA		
TAstV2-F-DC			ACCAACWGAGAAAGARCTGCA	75 bp	
TAstV2-F-DP			TTRCGGATGGAAAGAAGYCT		
ANV-F	UTR		GTAAACCACTGGCTGGCT	73 bp	[28]
ANV-R			TCCTGTACCCTCGATGCTA		
PanAstro F1	ORF1b	Hemi-nested PCR	GARTTYGATTGGRCKCGKTAYGA	~400 bp	[26]
PanAstro F2			GARTTYGATTGGRCKAGGTAYGA		
PanAstro R			GGYTTKACCCACATNCCRAA		
PanAstro HN F1			CGKTAYGATGGKACKATHCC		
PanAstro HN F2			AGGTAYGATGGKACKATHCC		

**Table 2:** Repeatability assay using ten-fold serial dilutions for TAstV-1, TAstV-2, and ANV. Cq: cycle of quantification; CV: coefficient of variation.

Copy Number	Inter-Assay						Intra-Assay					
	TAstV-1	TAstV-2	ANV	TAstV-1	TAstV-2	ANV	TAstV-1	TAstV-2	ANV	TAstV-1	TAstV-2	ANV
	Cq Mean			CV			Cq Mean			CV		
10^7^	13.6	12.48	13.14	0.25	0.2	0.41	13.49	12.49	12.76	0.19	0.17	0.67
10^6^	14.77	15.53	17.92	0.09	0.3	0.65	14.66	15.47	17.84	0.19	0.31	0.76
10^5^	18.18	25.17	20.8	0.23	0.99	0.57	18.03	24.99	19.81	0.17	0.96	0.9
10^4^	22.3	22.67	25.57	0.13	0.33	0.3	21.55	22.7	24.77	0.19	0.24	0.4
10^3^	24.86	26.09	29.11	0.22	0.22	0.55	24.89	26.26	28.24	0.39	0.26	0.47

**Table 3:** Positive samples of each turkey astrovirus based in age groups.

Detection of Turkey Astroviruses by RT-qPCR in Turkeys with Enteric Disease				
Age (Days)	TAstV-1	TAstV-2	ANV	Total Samples
≤10	1 (16%)	1 (16%)	6 (100%)	6 (3.0%)
>10 to ≤90	4 (3.0%)	59 (44%)	119 (89%)	134 (67%)
>90	4 (6.0%)	15 (25%)	53 (88%)	60 (30%)

**Table 4:** Turkey astroviruses gene copies (GCs) quantification divided by age groups in days in turkeys with enteric disease. GCs: gene copies; AV: average.

Quantification of Turkey Astrovirus by RT-qPCR							
Age Group (Days)	TAstV-1		TAstV-2		ANV		Total Samples
	GCs/mg	GCs/mg	GCs/mg	GCs/mg	GCs/mg	GCs/mg	
	AV	Max	AV	Max	AV	Max	
≤10	2.48 × 10^5^	2.48 × 10^5^	1.94 × 10^2^	1.94 × 10^2^	1.13 × 10^5^	4.22 × 10^5^	6
>10 to ≤90	1.46 × 10^6^	5.85 × 10^6^	1.30 × 10^10^	2.37 × 10^10^	8.28 × 10^8^	8.92 × 10^10^	134
>90	2.75 × 10^4^	9.00 × 10^4^	3.04 × 10^7^	8.37 × 10^8^	1.41 × 10^8^	7.34 × 10^9^	60

**Table 5:** Co-infections and single infections of turkey astroviruses (TAstV-1, TAstV-2, and ANV), showing the possible combinations of viral presence across samples and the corresponding number of positive cases for each combination. * indicates the most frequent co-infection observed in this study; # indicates the most frequent single infection observed in this study.

Turkey Astroviruses Co-Infections and Single Infections				
N° Combination	TAstV-1	TAstV-2	ANV	Total
1	x	x		0 (0.0%)
2	x		x	5 (2.7%)
* 3		x	x	63 (34%)
4	x	x	x	4 (2.0%)
5	x			0 (0.0%)
6		x		8 (4.3%)
# 7			x	106 (57%)

**Table 6:** Comparison of the nucleotide identities of partial ORF1b gene sequences of Ecuadorian samples of TAstV-1, TAstV-2, and ANV with other reference sequences. Sequences generated in this study are highlighted (Lilac = TAstV-2, Light blue = TAstV-1, Aqua green = ANV), whereas non-highlighted sequences correspond to strains retrieved from GenBank for comparison.

Sequence	1	2	3	4	5	6	7	8	9	10	11	12	13	14	15	16	17	18	19	20	21	22	23	24	25	26	27	28	Virus
**1. JX083371 CROATIA**	-	92.0	92.3	87.6	88.1	90.8	88.7	89.2	87.5	87.8	58.4	59.2	59.6	60.8	62.5	48.2	56.0	57.5	56.1	56.9	45.6	53.5	58.6	55.4	58.8	56.8	59.6	57.7	**TAstV-2**
**2. NC_005790 USA**	92.0	-	95.8	89.9	85.8	89.6	88.0	88.4	86.5	86.5	61.8	60.2	59.8	60.3	60.7	47.6	56.0	58.9	59.2	56.7	44.9	54.8	59.5	57.3	59.2	56.3	61.1	57.0	
**3. DQ381360 ITALY**	92.3	95.8	-	89.4	85.8	90.8	88.9	88.2	86.5	87.8	59.9	58.5	58.3	59.3	61.0	45.9	54.8	57.0	57.0	55.2	45.6	53.5	57.8	55.4	58.5	55.4	59.9	55.2	
**4. KM875548 TURKEY**	87.6	89.9	89.4	-	93.7	94.5	89.9	88.4	88.5	88.6	60.3	59.2	59.1	59.3	58.8	50.0	56.5	57.0	58.0	57.1	46.2	52.2	57.5	55.2	58.1	57.0	60.2	55.9	
**5. HQ317731 POLAND**	88.1	85.8	85.8	93.7	-	96.7	89.6	89.3	88.9	88.2	57.1	59.8	59.7	60.1	59.1	47.6	54.9	53.1	53.5	53.9	44.9	52.8	53.1	52.8	58.1	53.8	56.6	54.5	
**6. MH511538 SLOVAKIA**	90.8	89.6	90.8	94.5	96.7	-	90.8	90.5	88.0	89.3	58.4	59.0	58.9	58.3	59.5	46.8	56.9	56.8	57.3	57.1	48.4	52.2	55.8	54.2	57.7	55.3	58.8	56.3	
**7. EU143851 USA**	88.7	88.0	88.9	89.9	89.6	90.8	-	92.8	92.8	93.1	59.9	59.2	59.1	59.8	60.1	47.6	55.7	57.3	58.3	57.0	46.2	52.6	57.0	54.9	56.6	55.4	59.3	55.9	
**8. DQ066593 USA**	89.2	88.4	88.2	88.4	89.3	90.5	92.8	-	93.3	93.4	60.3	60.0	59.3	60.3	61.0	48.2	53.7	57.8	57.3	56.8	45.6	53.0	58.4	56.4	57.7	56.4	59.6	55.9	
**9. 78T ECU UDLA**	87.5	86.5	86.5	88.5	88.9	88.0	92.8	93.3	-	98.6	55.8	59.4	57.7	58.7	58.5	65.0	69.5	63.5	63.0	61.5	67.5	55.5	62.0	61.5	60.8	60.1	62.5	62.5	
**10. 72T ECU UDLA**	87.8	86.5	87.8	88.6	88.2	89.3	93.1	93.4	98.6	-	59.0	58.6	58.5	59.5	59.6	46.2	55.3	58.1	59.2	56.8	47.7	53.5	59.5	56.5	57.0	56.0	60.5	55.9	
**11. 33T ECU UDLA**	58.4	61.8	59.9	60.3	57.1	58.4	59.9	60.3	55.8	59.0	-	87.3	86.8	87.1	84.2	47.8	65.5	58.4	59.9	59.9	49.3	55.0	58.6	58.4	56.5	57.1	58.6	59.3	**TAstV-1**
**12. KU711047 BRAZIL**	59.2	60.2	58.5	59.2	59.8	59.0	59.2	60.0	59.4	58.6	87.3	-	95.8	91.0	88.7	46.3	56.7	55.2	54.2	54.2	47.5	55.9	54.8	54.9	58.2	54.4	57.2	58.0	
**13. KU711045 BRAZIL**	59.6	59.8	58.3	59.1	59.7	58.9	59.1	59.3	57.7	58.5	86.8	95.8	-	88.4	86.3	46.3	57.6	55.6	54.3	54.0	46.8	56.1	54.1	54.5	58.0	53.5	58.3	57.9	
**14. NC_002470 USA**	60.8	60.3	59.3	59.3	60.1	58.3	59.8	60.3	58.7	59.5	87.1	91.0	88.4	-	93.2	44.6	59.8	55.7	56.0	55.9	45.6	54.8	56.3	55.7	58.6	56.0	57.8	57.5	
**15. KF736884 POLAND**	62.5	60.7	61.0	58.8	59.1	59.5	60.1	61.0	58.5	59.6	84.2	88.7	96.3	93.2	-	42.3	58.5	55.7	55.4	55.2	44.9	54.3	56.2	57.0	60.6	55.1	59.2	55.6	
**16. JF815368 CROATIA**	48.2	47.6	45.9	50.0	47.6	46.8	47.6	48.2	65.0	46.2	47.8	46.3	46.3	44.6	42.3	-	84.4	86.8	83.2	84.1	85.2	77.2	80.5	84.4	86.9	85.0	89.4	87.3	**ANV**
**17. MK975886 BANGLADESH**	56.0	56.0	54.8	56.5	54.9	56.9	55.7	53.7	69.5	55.3	65.5	56.7	57.6	59.8	58.5	84.4	-	88.2	91.0	89.8	90.3	80.3	82.7	84.9	85.6	86.1	86.5	89.4	
**18. OM469237 SWITZERLAND**	57.5	58.9	57.0	57.0	53.1	56.8	57.3	57.8	63.5	58.1	58.4	55.2	55.6	55.7	55.7	86.8	88.2	-	92.2	90.5	88.4	81.3	86.6	87.5	88.0	86.1	89.7	90.5	
**19. OP967012 INDIA**	56.1	59.2	57.0	58.0	53.5	57.3	58.3	57.3	63.0	59.2	59.9	54.2	54.3	56.0	55.4	83.2	91.0	92.2	-	92.7	91.6	80.4	86.1	85.8	86.1	85.8	88.1	91.3	
**20. KU711049 BRAZIL**	56.9	56.7	55.2	57.1	53.9	57.1	57.0	56.8	61.5	56.8	59.9	54.2	54.0	55.9	55.2	84.1	89.8	90.5	92.7	-	92.9	79.1	83.8	84.4	83.5	84.9	87.8	89.5	
**21. HM755447 CROATIA**	45.6	44.9	45.6	46.2	44.9	48.4	46.2	45.6	67.5	47.7	49.3	47.5	46.8	45.6	44.9	85.2	90.3	88.4	91.6	92.9	-	77.2	83.1	83.9	86.9	85.2	85.9	91.0	
**22. 39T ECU UDLA**	53.5	54.8	53.5	52.2	52.8	52.2	52.6	53.0	55.5	53.5	55.0	55.9	56.1	54.8	54.3	77.2	80.3	81.3	80.4	79.1	77.2	-	92.8	79.1	83.0	82.2	80.9	81.5	
**23. 113T ECU UDLA**	58.6	59.5	57.8	57.5	53.1	55.8	57.0	58.4	62.0	59.5	58.6	54.8	54.1	56.3	56.2	80.5	82.7	86.6	86.1	83.8	83.1	92.8	-	86.3	90.0	90.5	86.5	87.5	
**24. KT355289 USA**	55.4	57.3	55.4	55.2	52.8	54.2	54.9	56.4	61.5	56.5	58.4	54.9	54.5	55.7	57.0	84.4	84.9	87.5	85.8	84.4	83.9	79.1	86.3	-	91.4	88.7	87.8	89.1	
**25. 166T ECU UDLA**	58.8	59.2	58.5	58.1	58.1	57.7	56.6	57.7	60.8	57.0	56.5	58.2	58.0	58.6	60.6	86.9	85.6	88.0	86.1	83.5	86.9	83.0	90.0	91.4	-	91.0	87.6	88.9	
**26. KT355284 USA**	56.8	56.3	55.4	57.0	53.8	55.3	55.4	56.4	60.1	56.0	57.1	54.4	53.5	56.0	55.1	85.0	86.1	86.1	85.8	84.9	85.2	82.2	90.5	88.7	91.0	-	87.2	90.5	
**27. PANA25 ECU UDLA**	59.6	61.1	59.9	60.2	56.6	58.8	59.3	59.6	62.5	60.5	58.6	57.2	58.3	57.8	59.2	89.4	86.5	89.7	88.1	87.8	85.9	80.9	86.5	87.8	87.6	87.2	-	87.5	
**28. PANA21 ECU UDLA**	57.7	57.0	55.2	55.9	54.5	56.3	55.9	55.9	62.5	55.9	59.3	58.0	57.9	57.5	55.6	87.3	89.4	90.5	91.3	89.5	91.0	81.5	87.5	89.1	88.9	90.5	87.5	-	

## Data Availability

All data are available in the manuscript and Supplementary Material.

## Supplementary Materials

The following supporting information can be downloaded at https://www.mdpi.com/article/10.3390/v18030308/s1, DATA—Table S1.

## Abbreviations

The following abbreviations are used in this manuscript:

AGROCALIDAD	Agency of Regulation and Control of Phytosanitary and Animal Health of Ecuador
ANV	Avian Nephritis Virus
AV	Average
Bp	Base pairs
CAstV	Chicken Astrovirus
cDNA	Complementary DNA
ChPV	Chicken Parvovirus
CV	Coefficient of Variation
Cq	Cycle of Quantification
ddH_2_O	Double-Distilled Water
DNA	Deoxyribonucleic Acid
*E. coli*	*Escherichia coli*
GDP	Gross Domestic Product
GCs	Gene Copies
Kb	Kilobases
LoD	Limit of Detection
LoQ	Limit of Quantification
LTS	Light Turkey Syndrome
MIQE	Minimum Information for Publication of Quantitative Real-Time PCR Experiments
NCBI	National Center for Biotechnology Information
NPV	Negative Predictive Value
NT	Nucleotide
NTC	Non-Template Control
ORF	Open Reading Frame
PBS	Phosphate Buffered Saline
PEC	Poult Enteritis Complex
PEMS	Poult Enteritis and Mortality Syndrome
PES	Poult Enteritis Syndrome
PPV	Positive Predictive Value
rcf	Relative Centrifugal Force
RNA	Ribonucleic Acid
RSS	Runting–Stunting Syndrome
RT-PCR	Reverse Transcription Polymerase Chain Reaction
RT-qPCR	Reverse Transcription Quantitative Polymerase Chain Reaction
TAstV	Turkey Astrovirus
TAstV-1	Turkey Astrovirus type 1
TAstV-2	Turkey Astrovirus type 2
TCoV	Turkey Coronavirus
TN	True Negatives
TP	True Positives
TuPV	Turkey Parvovirus
U.S.	United States
UDG	Uracil-DNA Glycosylase
UTR	Untranslated Region
WOAH	World Organization for Animal Health
